# Associations between cardiac and pulmonary involvement in patients with juvenile dermatomyositis—a cross-sectional study

**DOI:** 10.1007/s00296-021-05071-3

**Published:** 2022-01-04

**Authors:** Birgit Nomeland Witczak, Thomas Schwartz, Zoltan Barth, Eli Taraldsrud, May Brit Lund, Trond Mogens Aaløkken, Berit Flatø, Ivar Sjaastad, Helga Sanner

**Affiliations:** 1 Institute for Experimental Medical Research and KG Jebsen Center for Cardiac Research, Oslo University Hospital and University of Oslo, Oslo, Norway; 2 Department of Immunology, Oslo University Hospital, Rikshospitalet, Oslo Norway; 3 Institute of Clinical Medicine, Faculty of Medicine, University of Oslo, Oslo, Norway; 4 Oslo New University College, Oslo, Norway; 5 Department of Respiratory Medicine, Oslo University Hospital, Rikshospitalet, Oslo Norway; 6 Department of Radiology, Oslo University Hospital, Rikshospitalet, Oslo Norway; 7 Department of Rheumatology, Oslo University Hospital, Rikshospitalet, Oslo Norway; 8 Department of Cardiology, Oslo University Hospital, Ullevål, Oslo Norway

**Keywords:** Juvenile dermatomyositis/polymyositis, Cardiovascular disease, Lung disease, Echocardiography, Pulmonary fibrosis

## Abstract

This study aimed at exploring the association between detectable cardiac and pulmonary involvement in long-term juvenile dermatomyositis (JDM) and to assess if patients with cardiac and pulmonary involvement differ with regard to clinical characteristics. 57 JDM patients were examined mean 17.3 (10.5) years after disease onset; this included clinical examination, myositis specific/associated autoantibodies (immunoblot), echocardiography, pulmonary function tests and high-resolution computed tomography. Cardiac involvement was defined as diastolic and/or systolic left ventricular dysfunction and pulmonary involvement as low diffusing capacity for carbon monoxide, low total lung capacity and/or high-resolution computed tomography abnormalities. Patients were stratified into the following four groups: (i) no organ involvement, (ii) pulmonary only, (iii) cardiac only, and (iv) co-existing pulmonary and cardiac involvement. Mean age was 25.7 (12.4) years and 37% were males. One patient had coronary artery disease, seven had a history of pericarditis, seven had hypertension and three had known interstitial lung disease prior to follow-up. There was no association between cardiac (10/57;18%) and pulmonary (41/57;72%) involvement (*p* = 0.83). After stratifying by organ involvement, 21% of patients had no organ involvement; 61% had pulmonary involvement only; 7% had cardiac involvement only and 11% had co-existing pulmonary or cardiac involvement. Patients with co-existing pulmonary or cardiac involvement had higher disease burden than the remaining patients. Patients with either cardiac or pulmonary involvement only, differed in clinical and autoantibody characteristics. We found no increased risk of developing concomitant cardiac/pulmonary involvement in JDM. Our results shed light upon possible different underlying mechanisms behind pulmonary and cardiac involvement in JDM.

## Introduction

Juvenile dermatomyositis (JDM) is a rheumatic disease of childhood that mainly targets skin and muscles, but may also affect internal organs, such as the heart and lungs [1]. Clinical manifest lung disease, including interstitial lung disease (ILD), is rare in JDM, but associates with high morbidity and mortality [2]. Our group previously reported a high frequency of mostly subclinical pulmonary involvement (PI) in JDM patients comprehensively examined after median 16.8 years disease duration [3]. It is not known if these patients later will develop manifest lung disease. Although very rare, serious cardiac involvement (CI) has been reported in JDM [4]. However, in our cohort, subclinical CI is relatively frequent in patients with medium- to long-term JDM [5, 6], also found in patients after median 4.6y disease duration [7]. It is not known if patients with JDM later will develop manifest cardiac disease.

The coexistence of CI and PI has become more acknowledged during recent years; in the general population, subclinical impairment of lung function is related to mild cardiac dysfunction [8]. This relationship is also found in adults with connective tissue diseases, where ILD is associated with cardiovascular complications [9]. However, it is unknown whether JDM patients with PI are at higher risk of developing cardiac dysfunction and vice versa. Thus, the aim of our study was to explore the association between CI and PI in JDM patients; also, to assess if patients with CI and/or PI differ with regard to clinical characteristics.

## Methods

### Patients

Inclusion criteria were a probable/definitive diagnosis of DM [10], disease onset before 18y, minimum 24 m disease duration and age ≥ 6y. There were no exclusion criteria. A retrospective inception cohort of Norwegian JDM patients (diagnosed between 1970 and 2006, *n* = 59) was identified [11]. Patients with complete cardiac *and* pulmonary data (for details see below) were included in the present study. Informed consents were obtained from patients (if > 16 years) or their parents (if ≤ 16 years) at the time of follow-up examination. The study was approved by the regional committee of health and medical research ethics in South-East Norway (S-05144) (Aug 2005—June 2023).

### Data collection

Clinical examination was performed at a comprehensive *follow-up visit* of the identified patients at Oslo University Hospital from September 2005 to May 2009. In addition to lung and cardiac measures (see below), the examination included muscle strength/endurance measured by manual muscle test (MMT-8)/child myositis assessment scale (CMAS) and health-related quality of life (HRQOL) by the Short Form-36 physical and mental component summary scores (SF-36 PCS and MCS) in patients > 13y [12]. Cumulative prednisolone dose was calculated [11]. Inactive disease was defined according to the revised PRINTO criteria [13]. Nailfold capillary density was assessed as previously described [14]. Disease activity and cumulative organ damage were assessed at the follow-up visit and also scored retrospectively (by chart review) at 1 year post diagnosis using the Disease Activity Score for JDM (DAS) and the Myositis Damage Index (MDI), respectively, as previously described [11, 15].

### Cardiac measures

Two-dimensional, M-mode, and Doppler echocardiography was performed at the follow-up visit as previously described [5, 6]. Low early diastolic tissue velocity (e’) reflects left ventricular (LV) diastolic dysfunction [16] and low long axis strain (LAS) reflects LV systolic dysfunction [6]. *Cardiac involvement* was defined as LV diastolic or systolic dysfunction; both defined as mean of control subjects – 2SD from one of our previous studies [6]. Heart rate variability (expressed as cSDNN) was measured by Holter ECG [17]. Total cholesterol (TC) and high-density lipoprotein (HDL) were analyzed according to hospital routine; TC:HDL ratio was calculated (superior to other lipid parameters in predicting ischemic heart disease [18].

### Pulmonary measures

Pulmonary function tests (PFTs) were performed at the follow-up visit as previously described in detail [3]. For the present study, diffusing capacity for carbon monoxide (DLCO) (adjusted for Hgb) and total lung capacity (TLC) (both expressed as percentage of predicted) were included. Low TLC and DLCO were defined as less than the fifth percentile of the predicted values [19]. High-resolution computer tomography (HRCT) was carried out in all patients; HRCT-detected abnormalities includes ILD (reticular pattern, ground glass opacity) and airways disease (bronchiectasis, air trapping, micronodules) [3]. *Pulmonary involvement* was defined as low TLC, low DLCO, or HRCT- detected abnormalities.

### Stratification of patients

We stratified our patients into four groups based on the presence of detectable CI and PI at follow-up as follows: *group 1:* neither PI nor CI; *group 2:* PI only; *group 3:* CI only; and, *group 4:* co-existing PI and CI.

### Immunological analyses

Sera were thawed at the follow-up visit and later (2014) subjected to the following tests: (a) ANA screening by indirect immunofluorescence (IIF) on HEp-2 cells at serum dilution 1:160. Only nuclear fluorescence patterns were considered positive. Detection of myositis specific autoantibodies (MSA) and myositis associated autoantibodies (MAA) [20] using (b) the myositis line immunoassay A1 which included: MDA5, TIF1-γ, NXP-2, SAE1, SAE2, HMGCR-S (Sigma), HMGCR-E (EUROIMMUN AG), Mup44 and (c) the myositis line immunoassay A2 (Myositis Profile 3 Euroline) which included: Jo1, PL-7, PL-12, EJ, OJ, SRP, Mi-2, PM-Scl75, PM-Scl-100 Ku and Ro52; both from Euroimmun AG Lübeck, Germany. The immunoblot strips were scanned and evaluated digitally, using the Euroline scan. Signal intensities below 11 were regarded as negative.

### Statistical analysis

Data were expressed as mean (SD), median (IQR) or numbers (%) as appropriate. Differences between patient groups were tested by one-way ANOVA with Tukey post-hoc test, Kruskal–Wallis test with Dunn post-hoc test or Chi-square (no post-hoc test applied due to small numbers). Due to the hypothesis generating nature of our study, we did not correct for multiple comparisons. Correlations were determined by the Spearman correlation coefficient (r_sp_). Chi-square goodness of fit test was used to explore whether the number of patients in the clinical groups were as predicted based on the fraction with CI and PI in our cohort. Due to small numbers, no statistics was performed for MSA/MAA between groups. IBM SPSS Statistics v. 25.0 (IBM, Armonk, NY, USA) was used for statistical analyses.

## Results

### Clinical characteristics

The JDM cohort included 57 patients, representing 97% of the eligible patients. One patient had coronary artery disease, seven had pericarditis during disease course, seven had hypertension and three had known ILD prior to follow-up. There were no cases of diabetes, heart failure or cardiac arrhythmia. In total, 10/57 (18%) of patients had CI and 41/57 (72%) had PI; the organ involvement was mostly subclinical.

### Associations between CI and PI

**Fig. 1:**
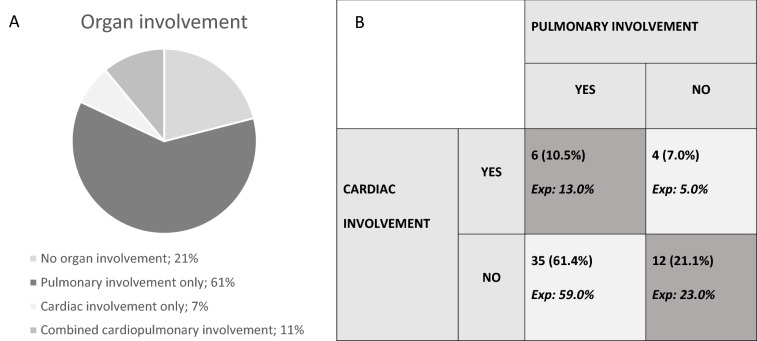
**A** Distribution of patients in the four clinical groups; **B** Distribution of patients in the four clinical groups compared to expected frequencies based the fraction with lung and cardiac involvement in our cohort; *Exp* expected distribution; *p* = 0.83 (Chi-square goodness of fit test)

After stratification into groups, *group 1 (*neither PI nor CI) consisted of 12(21%) patients; *group 2* (PI only) 35(61%); *group 3:* (CI only) 4(7%) and, *group 4:* co-existing PI and CI 6(11%) (Fig. 1A) (Table 1). No associations between CI and PI were found when comparing with the expected distribution (Fig. 1B, *p* = 0.83). We then correlated the variables defining PI (TLC, DLCO as continuous variables and HRCT abnormalities) and variables defining CI (e´ and LAS as continuous variables) with each other; no significant correlations were found (data not shown).

**Table 1:** Patient and disease characteristics and clinical variables stratified across the four groups

	Patients total	Group 1 No organ involvement	Group 2 Pulmonary only	Group 3 cardiac only	Group 4 coexisting pulmonary and cardiac	*Significant p-values* (between groups)
Number of pts, *n* (%)	57 (100)	12 (21)	35 (61)	4 (7)	6 (11)	NA
Age at follow-up, *y*	25.7 (12.4)	26.2 (11.9)	22.5 (11.3)	35.2 (7.6)	37.7 (13.5)	2 vs 4: *p* = 0.021
Disease duration, *y*	17.3 (10.5)	16.9 (11.5)	14.7 (9.1)	28.5 (5.2)	25.5 (11.7)	2 vs 3: *p* = 0.047
Male sex, *n* (%)	21 (37)	2(17)	11 (31)	3 (75)	5 (83)	*P* = 0.014^*b*^
Smokers daily disease course; *n* (%)^a^	14 (30)	2 (18)	7 (27)	1 (25)	4 (67)	*NS*
DAS 1 y	5.9 (3.9)	5.9 (4.2)	4.8 (3.5)	10.1 (4.1)	9.0 (3.0)	2 vs 3: *p* = 0.039
MDI 1 y	1.0 (0.0–2.0)	1.0 (0.0–2.5)	0.0 (0.0–2.0)	2.5 (1.5–3.5)	2.0 (1.0–5.0)	*NS*
Inactive Disease, *n* (%)	28 (49)	6 (50)	18 (51)	3 (75)	1 (17)	*NS*
Calcinosis, *n* (%)	21 (37)	5 (42)	10 (29)	2 (50)	4 (67)	*NS*
Lipodystrophy, *n* (%)	10 (18)	1 (8)	2 (6)	2 (50)	5 (83)	*P* < *0.001 *^*b*^
Hypertension, disease course, *n* (%)	7 (12)	1(8)	2 (6)	2 (50)	2 (33)	*P* = *0.025 *^*b*^
TC:HDL ratio *N* = 50	3.9 (2.0)	3.1 (0.4)	3.5 (1.3)	7.4 (3.2)	5.5 (2.8)	1 vs 3: *p* < 0.001 1 vs 4: *p* = 0.044 2 vs 3: *p* < 0.001 2 vs 4: *p* = 0.049
HRV, cSDNN, *N* = 55	39.7 (16.7)	50.5 (21.8)	36.8 (13.6)	42.7 (2.3)	29.5 (17.6)	1 vs 4: *p* = 0.050
NCD, cap/mm	6.4 (2.1)	7.1 (1.8)	6.1 (2.3)	7.5 (0.6)	6.4 (2.1)	*NS*
Pred/DMARD, *n* (%)	17 (30)	4 (33)	10 (29)	1 (25)	2 (33)	*NS*
Cum Prednisolone during disease course, g	7.9 (3.6–12.6)	8.9 (7.6–11.3)	4.8 (2.5–10.6)	17.9 (12.6–26.6)	14.4 (7.9–27.3)	2 vs 3; *p* = 0.008 2 vs 4; *p* = 0.005
DAS	4.7 (3.0)	4.0 (2.4)	4.5 (2.8)	3.4 (2.5)	8.5 (2.8)	1 vs 4: *p* = 0.008 2 vs 4: *p* = 0.008 3 vs 4: *p* = 0.025
MDI	4.3 (3.1)	4.3 (2.2)	3.5 (2.8)	5.2 (3.3)	8.2 (3.8)	1 vs 4: *p* = 0.036 2 vs 4: *p* = 0.003
MMT-8	76.5 (4.7)	77.2 (3.1)	76.7 (4.1)	80.0 (0.0)	71.3 (8.5)	1 vs 4: *p* = 0.048 2 vs 4: *p* = 0.040 3 vs 4: *p* = 0.019
CMAS	48.3 (5.4)	48.8 (3.2)	48.8 (4.7)	51.0 (1.4)	42.8 (10.5)	*NS*
SF-36, PCS ^a^	50.8 (9.0)	51.0 (9.7)	52.3 (7.3)	56.0 (3.4)	40.2 (10.9)	2 vs 4: *p* = 0.011 3 vs 4: *p* = 0.023
SF-36, MCS^a^	53.4 (7.7)	51.8 (6.4)	53.6 (8.1)	52.7 (5.7)	55.4 (10.1)	*NS*

Variables are assessed at follow-up if not otherwise stated; values are mean (SD) or median (25th—75th percentile) if not otherwise stated; *NA *not assessed, *NS*  non-significant, *DAS* Disease activity score, *MDI*  myositis damage index, *FU*  follow-up, *TC*  Total cholesterol, *HDL*  high-density lipoprotein, *NCD*  nailfold capillary density, *HRV*  heart rate variability, *NCD*  Nail fold capillary density, *DMARD*  disease modifying antirheumatic drugs, *CMAS*  child myositis assessment scale, *SF-36*  Short Form-36, *PCS*  physical assessment scale, *MCS*  mental component scale, Post-hoc tests not run for categorical variables due to low *n* in several groups. ^a^assessed in patients ≥ 14y at follow-up, *n* = 47; ^b^post hoc tests not assessed

### Variables defining CI and PI across the four clinical groups

**Fig. 2:**
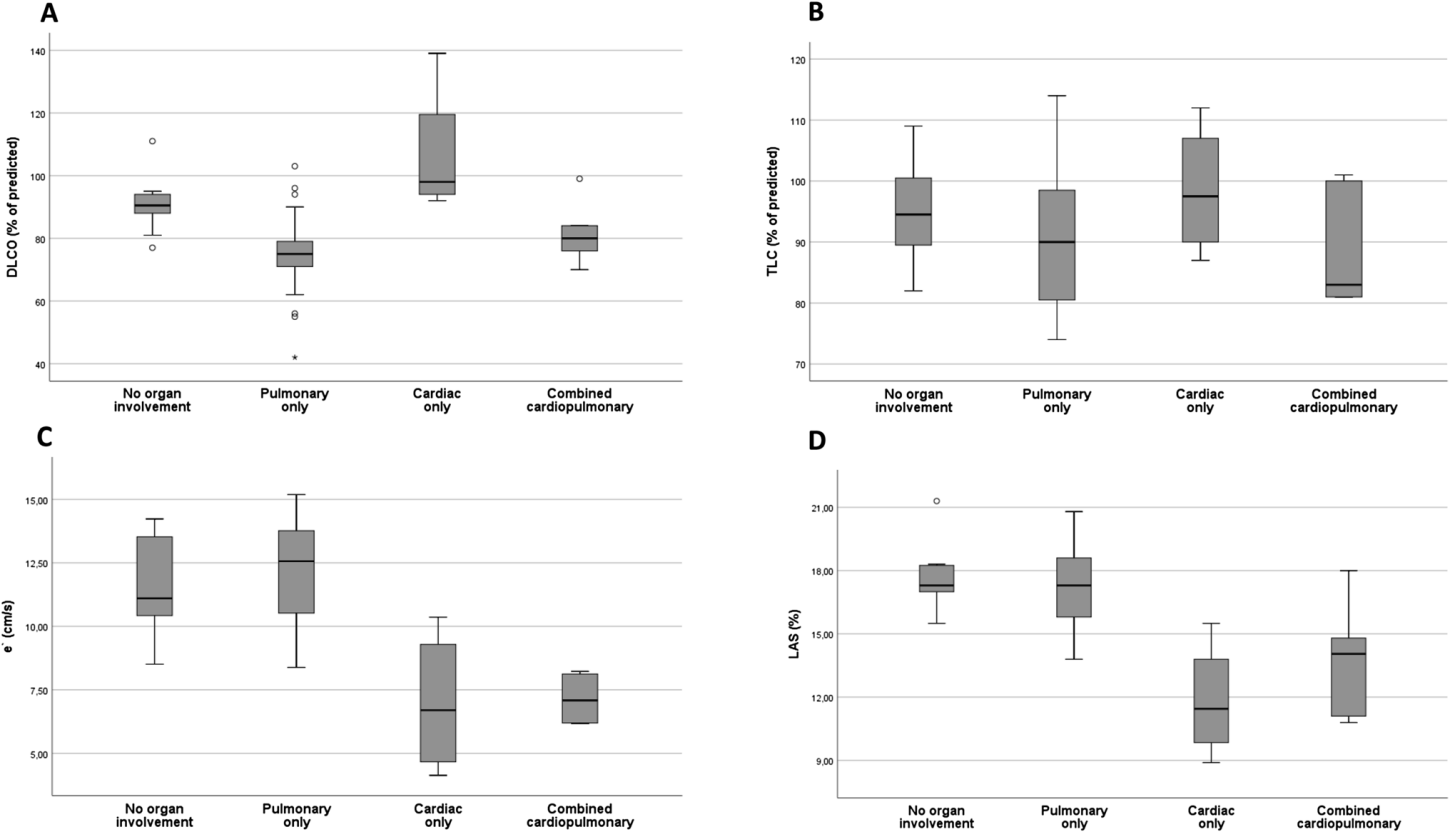
Variables defining cardiac and pulmonary involvement across the four clinical groups; p-values based on one-way ANOVA with Tukey post-hoc tests; **A** DLCO: Diffusing capacity for carbonmonoxide, % of predicted; **B** TLC: Total lung capacity; % of predicted; **C** e´: early diastolic tissue velocity, cm/s; **D** LAS: long-axis strain, %

Naturally, DLCO was lower in group 2 and 4 (which include patients with PI) compared with the other two groups (*p*’s < 0.007) except for group 1 vs 4, NS) (Fig. 2A). For TLC, there were similar, albeit non-significant trends (Fig. 2B). Naturally, e´ and LAS were lower in group 3 and 4 (which include patients with CI) compared with the other two groups (*p*’s < 0.002) (Fig. 2C and D).

### Clinical variables across the four clinical groups

Group 4 (co-existing CI and PI) differed the most from the other groups; they were older at follow-up (only statistically significant compared to group 2), had numerically more males (5/6, NS), and 4/6 were daily smokers (Table 1). Also, group 4 had higher DAS at follow-up compared with the remaining sub-groups, as well as higher MDI than group 1 and 2. Group 4 had numerically more calcinosis, lipodystrophy as well as higher TC:HDL ratio compared with groups 1 and 2. They also had lower heart rate variability (cSDNN) than group 1. Group 4 also had more impaired SF-36 PCS than both groups 2 and 3. Group 3 (CI only) had higher early DAS, longer disease duration and higher cumulative prednisolone dose compared with group 2; also, they had higher TC:HDL ratio compared to both group 1 and 2. On the other hand, group 3 had less impaired SF-36 PCS than group 2 (Table 1).

MSA and MAA are shown in Table 2 as background data. 31(54%) had no detectable ANA. 19(33%) of patients had at least one detectable MSA/MAA; of those 13(68%) had one autoantibody and 6(32%) had > 1 autoantibody (Table 2). MSA known to be associated with lung involvement (PL-7, Jo-1 and MDA5) were only found in group 2 (PI only).

**Table 2:** Myositis specific- and myositis associated autoantibodies stratified across the four JDM groups

	Patients total	Group 1 no organ involvement	Group 2 pulmonary only	Group 3 cardiac only	Group 4 coexisiting pulmonary and cardiac
*n*	57	12	35	4	6
ANA IIF	26 (46)	6 (50)	16 (46)	1 (25)	3 (50)
No MSA/ MAA	38 (67)	7 (58)	23 (66)	3 (75)	5 (83)
***Myositis specific autoantibodies***					
Jo-1	1 (2)	0	1 (3)	0	0
PL-7	1 (2)	0	1 (3) ^4^	0	0
SRP	1 (2)	1 (8)	0	0	0
Mi-2	3 (5)	1 (8)^1^	2 (6)	0	0
NXP-2	5 (9)	1 (8)^3^	3 (9)^4^	0	1 (25)
TIF1- γ	1 (2)	0	0	1 (25)	0
MDA5	2 (4)	0	2 (6)^5^	0	0
SAE-1	1 (2)	1 (8)^2^	0	0	0
HMGCR	1 (2)	1 (8)^1^	0	0	0
***Myositis associated autoantibodies***					
PMScl75	3 (5)	0	3 (9)^6^	0	0
PMScl100	2 (4)	0	2 (6)^5^	0	0
Ku	2 (4)	1 (8)	1 (3)	0	0
Ro52	3 (5)	2 (17) ^2,3^	1 (3) ^6^	0	0

*MSA* Myositis specific autoantibodies, *MAA* Myositis associated autoantibodies, Numbers are *n* (%); No patients had detectable EJ, PL-12, OJ, SAE-2 or Mup44; ^1,2,3,4,and 5^denotes that more than one MSA and/or MAA are present in the same patient. *IIF* indirect immunofluorescence

## Discussion

Based on previous studies in the general population [8] and connective tissue disease [9], we hypothesized that there was an increased risk of concomitant detectable cardiac and pulmonary involvement in JDM. No evidence for a higher risk of CI given PI and vice versa was detected in our study. However, due to small sample size our results should be interpreted with caution.

Special attention should be drawn to group 4 (combined CI and PI). Despite being small (*n* = 6/11% of the total cohort), several trends and statistically significant differences were found when compared with the other groups. This group displayed a higher disease burden (including higher disease activity and damage and also unfavorable lipid profile and heart rate variability) and experienced a reduced quality of life (SF-36 PCS) compared with the other groups. They were characterized by older age, more likely to be males and daily smokers. Importantly, the high MDI observed in this group is not explained by subclinical PI and CI.

Second, group 3 (CI only) had higher disease activity at 1 year post-diagnosis, unfavorable lipid profile, and higher cumulative prednisolone dose than group 2 (PI only). The higher age found in patients with cardiac involvement might reflect that they were diagnosed in a time with less aggressive treatment; also, it is known that cardiac involvement increases with age.

Interestingly, the patients with CI, either isolated (group 3) or combined (group 4) bear little resemblance with the patients with PI. Possibly, abnormalities in the heart and lung may represent two different mechanisms/phenotypes. This may explain the lack of correlation between subclinical heart and lung involvement in JDM patients. Although no statistical difference in NCD across clinical groups was found in the present study, we have previously reported an association between microvascular findings and PI, but no association between microvascular findings and CI [14].

Data on MSA and MAA was used as background information. 67% has no detectable MSA or MAA which is lower than reported in the literature [20]. This might be explained by methodological differences between assay methods [21]. Interestingly, MSA known to be associated with pulmonary involvement in JDM (MDA5, PL-7 and Jo-1)[20], were only found in patients with PI.

Strengths of our study include that our patients come from a restrospective inception cohort and they were comprehensively examined after medium- to long term follow-up. Limitations include that we did not calculate sample size (due to the hypothesis-generating nature of our study) and the cross-sectional design.

To conclude, no increased risk for concomitant heart and lung involvement was found in JDM. Our results may suggest different mechanisms underlying pulmonary and cardiac involvement in JDM; this requires further study. Based on our results, clinicians should be especially aware of the risk detectable organ involvement in patients with high disease activity and damage after medium- to long-term disease. Follow-studies are needed to see if patients with subclinical PI and CI later develop clinical manifest lung and/or cardiac disease.

## Statement on open data sharing

The data will not be deposited, due to guidelines from the regional committee of health and medical research ethics in South-East Norway.
